# When Bone Deforms the Aorta: Long-Term Vascular Remodeling Secondary to Juvenile Osteoporosis

**DOI:** 10.7759/cureus.110979

**Published:** 2026-06-16

**Authors:** José Miguel Zermeño, Deneisy H Trinidad Cruz, Gloria Jimena Coello, Diana V Jimenez Peña, Eva Brittania Delgado

**Affiliations:** 1 Surgery, General Hospital of the Institute for Social Security and Services for State Workers (ISSSTE), Aguascalientes, MEX; 2 General Surgery, General Hospital of the Institute for Social Security and Services for State Workers (ISSSTE), Aguascalientes, MEX; 3 Internal Medicine, General Hospital of the Institute for Social Security and Services for State Workers (ISSSTE), Ciudad Juarez, MEX

**Keywords:** aortic deformation, case report, ct angiography, juvenile osteoporosis, kyphoscoliosis

## Abstract

Pregnancy- and juvenile-associated osteoporosis is a rare condition that may lead to pathological fractures and severe vertebral deformities throughout life. We present the case of an 84-year-old female with a history of osteoporosis, diagnosed by dual-energy X-ray absorptiometry (DXA) during her first pregnancy. Over a period of more than six decades, she sustained multiple osteoporotic vertebral compression fractures of the T12, L1, and L3 vertebral bodies, leading to progressive kyphoscoliosis and significant loss of height. During routine follow-up for chronic obstructive pulmonary disease, a computed tomography scan requested by the Pulmonology Department revealed marked elongation and deviation of the thoracoabdominal aorta secondary to the underlying spinal deformity. Given these findings, the patient was referred to the Angiology Department for further evaluation. This case highlights a rarely described interaction between long-standing musculoskeletal deformities and vascular anatomy, particularly involving displacement and remodeling of major vessels such as the aorta. These findings emphasize the importance of cross-sectional imaging and a multidisciplinary approach in the assessment of complex chronic conditions with multisystem involvement.

## Introduction

Pregnancy- and lactation-associated osteoporosis (PLO) is a particularly rare form of osteoporosis that typically occurs during the third trimester of pregnancy or the postpartum period. It was first described by Nordin and Roper in 1955. PLO is characterized by fragility fractures, most commonly involving the vertebral bodies, and severe back pain. The incidence of PLO is estimated to be approximately 0.4 per 100,000 women, although a significant number of cases likely remain undiagnosed due to its subtle presentation and overlap with common postpartum conditions including mechanical low back pain, musculoskeletal strain, sacroiliac joint dysfunction, pelvic girdle pain, and postpartum fatigue [1]. Its etiology remains incompletely understood, with proposed mechanisms including hormonal changes, vitamin D deficiency, and genetic predisposition [2]. Early-onset disease may result in multiple fractures, progressive axial deformity, and long-term disability.

The association between spinal deformities and major vascular alterations is uncommon. However, previous studies suggest that severe spinal curvature may alter the anatomical course of large vessels, particularly the thoracic and abdominal aorta [3].

## Case presentation

An 84-year-old female from Mexico City with a history of juvenile osteoporosis diagnosed at age 22 during her first pregnancy presented for evaluation.

Her medical history included multiple vertebral fractures (cervical, thoracic, and lumbar), as well as fractures of the femur, humerus, and hip. She had required a walker for ambulation for the past 15 years. Comorbidities include chronic obstructive pulmonary disease (COPD) of 20 years' duration, treated with tiotropium bromide inhalation solution (0.226 mg/mL; 4-mL device), administered as two inhalations once daily; systemic hypertension of 50 years' duration, treated with telmisartan/hydrochlorothiazide 40/12.5 mg orally every 12 hours; and hypothyroidism of 40 years' duration, treated with levothyroxine 100 μg orally from Monday through Friday. All comorbid conditions were adequately controlled under the current treatment regimen. On admission, her blood pressure was 130/85 mmHg, heart rate was 86 beats/min, respiratory rate was 19 breaths/min, and body temperature was 36.4°C. Thyroid function testing demonstrated adequate control of hypothyroidism, with a thyroid-stimulating hormone (TSH) level of 2.36 mIU/mL, total triiodothyronine (T3) of 0.65 ng/ml, total thyroxine (T4) of 7.29 μg/dL, and free thyroxine (free T4) of 1.2 ng/dL.

A computed tomography scan performed for COPD follow-up revealed an incidental finding of elongation and S-shaped deviation of the thoracoabdominal aorta, with marked angulation following the spinal curvature. Visceral vessels were patent, with no evidence of aneurysm or dissection (Figure 1).

**Figure 1:**
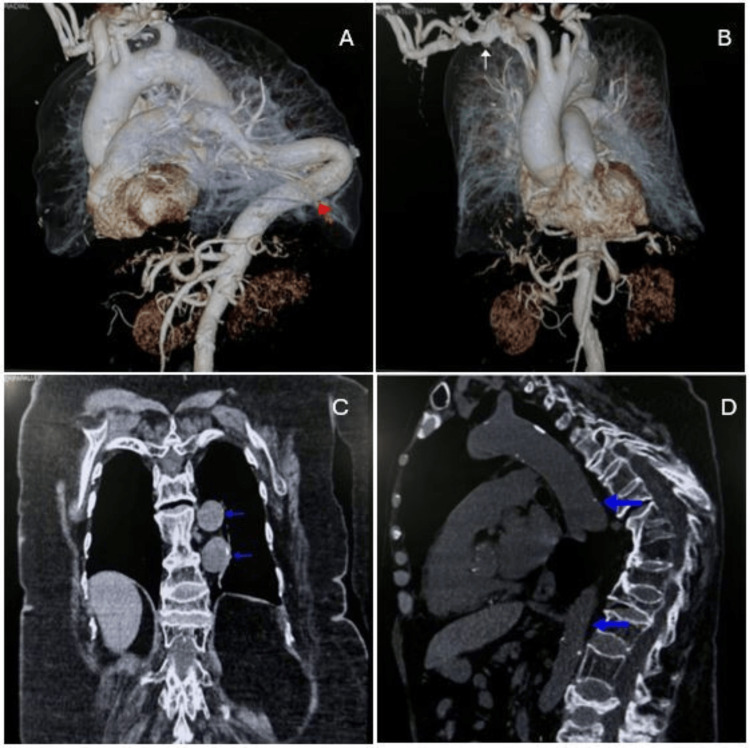
Computed tomography (CT) demonstrating marked elongation and tortuosity of the thoracoabdominal aorta with associated vascular abnormalities. (A) Oblique three-dimensional (3D) reconstruction showing elongation of the descending thoracic aorta (red arrow). (B) Frontal three-dimensional (3D) reconstruction demonstrating associated vascular anomalies (white arrow). (C) Coronal CT image showing apparent discontinuity of the descending aorta secondary to severe S-shaped angulation (blue arrows). (D) Sagittal CT image confirming the apparent discontinuity of the descending aorta due to marked S-shaped angulation (blue arrows).

The patient was referred to the Angiology and Vascular Surgery Department. During the comprehensive clinical evaluation, chronic pain was identified, associated with severe kyphoscoliosis. She reported progressive height loss from 1.59 m to 1.39 m.

Conservative management was implemented with an adequate clinical response. Conservative management, including strict blood pressure control, optimization of antihypertensive therapy, smoking cessation, management of cardiovascular risk factors, and annual imaging surveillance with computed tomography or magnetic resonance imaging, can successfully stabilize long-standing vascular and musculoskeletal deformities. In selected patients, this non-operative approach may effectively limit the progression of aortic remodeling, delay aneurysmal enlargement, and reduce the need for immediate surgical intervention [3].

The patient is currently undergoing multidisciplinary outpatient follow-up. Surgical interventions are discouraged due to the high risk of major complications secondary to the proximity of vital structures, particularly the thoracoabdominal aorta.

## Discussion

This case illustrates the complex interaction between long-standing skeletal deformities and secondary vascular adaptations [2]. Previous studies have demonstrated that aortic position may be altered in patients with degenerative scoliosis, leading to changes in vascular trajectory and angulation [4].

The Aortic Tortuosity Index (ATI) is a novel and reproducible quantitative measure of aortic tortuosity, calculated as the ratio between the aortic centerline length and the straight-line distance between its endpoints. Increased ATI has been associated with a more severe aortic phenotype and progressive aortic remodeling. In the present case, the ATI was calculated at 1.7, indicating marked aortic tortuosity and supporting the severity of the underlying anatomical alterations. Beyond its descriptive value, ATI may provide useful information for risk stratification, longitudinal surveillance, and planning of future surgical or endovascular interventions, particularly in patients with complex aortic anatomy. [5]

In this patient, severe kyphoscoliosis secondary to multiple fractures resulted in significant elongation and deviation of the aorta, a finding rarely reported in the literature. To our knowledge, significant long-term aortic remodeling secondary to juvenile osteoporosis has been rarely described. 

Additionally, studies on spinal deformity correction have shown that vertebral manipulation can increase aortic wall stress, particularly in elderly patients [6]. This finding may have important implications in surgical planning and risk assessment in patients with severe spinal deformities. Progressive aortic elongation may alter wall stress distribution and has been associated with an increased risk of aneurysm formation, aortic dissection, and rupture. Furthermore, excessive tortuosity can contribute to branch vessel malperfusion, thromboembolic events, and technical challenges during open surgical or endovascular interventions. Several studies suggest that aortic elongation may represent an additional risk factor beyond aortic diameter alone [7].

Management of patients with severe aortic elongation and tortuosity should be individualized according to symptoms, aortic dimensions, disease progression, anatomical characteristics, and overall surgical risk. Conservative management remains the preferred approach in clinically stable patients without indications for intervention. The principal advantages of this strategy are avoidance of procedural morbidity and preservation of quality of life [8]. Open surgical repair remains the definitive treatment for patients with significant aneurysmal degeneration, rapid aortic growth, symptomatic disease, or complications such as dissection and impending rupture [8].

Endovascular approaches, including thoracic endovascular aortic repair (TEVAR), offer a less invasive alternative and may reduce perioperative complications, hospital stay, and recovery time [8]. Nevertheless, marked aortic tortuosity and elongation can increase procedural complexity, compromise device deployment, and predispose to complications such as endoleaks, graft migration, and the need for secondary interventions. Therefore, careful anatomical assessment and multidisciplinary evaluation are essential when selecting the most appropriate therapeutic strategy [8].

Beyond anatomical considerations, there is increasing evidence of shared pathophysiological mechanisms between osteoporosis and vascular disease, including alterations in calcium metabolism and tissue remodeling [9]. Severe complications such as aortic injury during spinal procedures have also been described, further emphasizing the importance of understanding vascular anatomical variations in these patients [10].

## Conclusions

Juvenile osteoporosis may lead to long-term multisystem complications. This case demonstrates significant aortic remodeling secondary to severe spinal deformity, highlighting the importance of cross-sectional imaging and multidisciplinary management. Although not performed in the present case, three-dimensional magnetic resonance imaging may provide additional quantitative assessment of aortic tortuosity through calculation of the ATI. Awareness of vascular displacement in patients with severe spinal deformities is essential to reduce potential risks during diagnostic or surgical interventions.
